# On the Nature of the Spleen

**Published:** 1871-04

**Authors:** 


On the Nature of the Spleen.—Dr. Silvester, whose name is so well known as the promulgator of a method of resuscitating those apparantly dead from drowning, etc., believes that he has discovered the true nature of the spleen, from an investigation of the lateral homologies of the liver, stomach, intestinal canal. His pamphlet is long and learned, and illustrated by a somewhat fanciful diagram representing the "missing links" in our interior. The conclusion he arrives at may be given in his own words: "The spleen is a sanguiferous gland, situated on the left side of the abdominal cavity. It is the left lateral homologue of a portion of the liver, the liver being a combination of a sanguiferous gland and a biliary apparatus." The left biliary apparatus, the left end of the stomach, left pancreas and small intestines being all missing in our bodies as presently constituted, the only remains of the latter being the appendix vermiformis. The heroine of one of Disraeli's novels is represented as expressing her application of prevalent theories of development by the exclamation, "Oh!

I know, I was a fish, and I shall be a bird." But Dr. Silvester's views of the developmant of the spleen seem even more incomprehensible, and it is difficult to conceive how our knowledge of the spleen is to be advanced by their adoption, or why their promulgation should be entitled a "discovery."—*Pamphlet* published by John Churchill & Sons.—*Edinburgh Med. Journal.*

